# Optimizing climate change communication: Context Comparison Model method

**DOI:** 10.3389/fpsyg.2022.897460

**Published:** 2022-08-03

**Authors:** Viviane Seyranian, Doug Lombardi, Gale M. Sinatra, William D. Crano

**Affiliations:** ^1^Psychology Department, California State Polytechnic University, Pomona, Pomona, CA, United States; ^2^Department of Human Development and Quantitative Methodology, University of Maryland, College Park, College Park, MD, United States; ^3^Rossier School of Education and Psychology Department, University of Southern California, Los Angeles, CA, United States; ^4^Psychology Department, Division of Behavioral and Organizational Sciences, Claremont Graduate University, Claremont, CA, United States

**Keywords:** social identity, political party, minority influence, context comparison model, attitude change, climate change, science communication

## Abstract

*The Context Comparison Model* (CCM) provides a promising avenue to guide persuasive communication development by highlighting the features of the communication context that require consideration, including *source*, *target*, and *task* variables. The model was tested in a study of global climate change. American participants read a text outlining scientific evidence for global climate change and a policy proposal to mitigate future climate change. Prior to reading the text, participants’ completed measures of their political affiliation (Republican, Democrats, Independent or Other) to render their group memberships salient. They were randomly assigned to one of four source conditions: (a) ingroup minority; (b) ingroup majority; (c) outgroup minority; or (d) outgroup minority. Participants completed pre- and post-measures of attitudes and the plausibility of climate change. Pretest scores revealed that global climate change attitudes were held less strongly by Republicans than Democrats. In line with expectations, participants’ subjective attitudes were more influenced by ingroup sources, and larger persuasive effects were obtained for ingroup minorities. For the plausibility of climate change, participants were more persuaded by an outgroup source, and larger effects were evident for outgroup majorities. Results were precisely predicted by the CCM. Their implications for science communication were discussed.

## Optimizing climate change communication: The Context Comparison Model method

The scientific evidence supporting global climate change and its anthropogenic causes is now more robust and unanimous than ever in the scientific community (Intergovernmental Panel on Climate Change, 2021). Individuals also are more aware of global climate change than in the past. The vast majority (90%) of North Americans and Europeans now report awareness of climate change, yet concern is not on par with climate change awareness (Lee et al., 2015). Seventy percent of people surveyed from 40 countries indicated that they think climate change is a “very or extremely serious problem” (Andi and Painter, 2020). Additionally, poll after poll in the West shows that global climate change and policies to mitigate its effects are not top national priorities. For example, only 42% of Americans believe climate change should be the top priority for the president and Congress to address this year (Pew Research Center, 2022). A similar proportion (42%) of Europeans believe climate change should be a top governmental priority (Eurobarometer, 2022). Overall, these data underscore the fact that despite widespread scientific consensus on global climate change and scientists’ repeated calls for immediate action, most citizens do not view climate change as a foremost national priority even though the vast majority understand the serious nature of the problem.

This trend reflects the broader challenge of communicating scientific information to the public (Sinatra et al., 2014; Sinatra and Hofer, 2021). Scientific topics such as the health risks of tobacco use, COVID-19 vaccine effectiveness, and human induced climate change have accumulated enough scientific evidence to invoke a wide scientific consensus in the scientific community. However, this scientific consensus may not easily translate into public support. Instead, science-based issues often incite a full spectrum of public responses from acceptance and policy implementation to full-scale political controversies, misunderstandings, adamant denial, and distortions. Science communication requires navigating a complex social influence terrain where the audience often interprets scientific communications based on their interests, values, and group memberships (National Academies of Sciences, Engineering, and Medicine, 2017) rather than the quality of scientific evidence. Although science seeks to offer objective and evidence-based knowledge and solutions, the interpretation of scientific evidence by the public is often driven by subjective factors. Furthermore, increased science literacy does not necessarily translate into policy decisions and behavior change (e.g., Leiserowitz, 2006; Nisbet and Scheufele, 2009; Kahan, 2015). In fact, individuals high in scientific reasoning become more polarized based on group-based affiliations (Kahan, 2015), such as political orientation.

Although considerable research has examined climate change communication (Bråten et al., 2011; Jaeger and Wiley, 2015), complex scientific information related to climate change often has been communicated to the public with little attention to the psychological variables that may affect message receptivity. Insights from the social influence literature may be particularly beneficial in shedding light on effective strategies for communications on scientific issues such as global climate change. The *Context Comparison Model* (CCM; Crano and Alvaro, 1998; Crano, 2000, 2012; Crano and Seyranian, 2009) underlines the importance of considering a variety of variables to optimize communication. The current research tested predictions derived from the model in the context of the controversial science-based topic of global climate change.

## The Context Comparison Model

The CCM considers *source*, *target*, and *task* variables in the persuasion equation. It outlines optimal conditions for immediate, indirect, and delayed attitude change depending on the specific combination of target *attitude types* (weak or strong), *source types* (ingroup or outgroup status, minority or majority status), and *the nature of the issue* (subjective or objective). In this research, we consider these variables in detail and their relevance for controversial issues.

### Attitude formation versus attitude change

A central consideration in the CCM is the target’s attitude prior to the influence attempt. *Attitude formation* contexts involve attitudes that are weak, undeveloped, ambivalent, or non-existent. *Attitude change* contexts involve strong attitudes held with conviction. According to the CCM, in attitude change contexts, strong attitudes are resistant to influence, but are susceptible to change of related attitudes (indirect attitude change) if the source is an in-group minority. In attitude formation contexts, weak attitudes are more susceptible to direct attitude change, if the persuasive message is attributed to an in-group minority.

The attitude formation versus attitude change distinction is highly relevant in global climate change communication. We surmise that many individuals have developed attitudes about climate change, but they may be weak or ambivalent. This may be because the science behind global climate change is complex and is often perceived as uncertain and controversial (Shackley and Wynne, 1996). It also may be related to the fact that global climate change concerns events that may or may not transpire or directly influence individuals. Often, there is an element of ambiguity and unpredictability associated with global climate change that may spark defensive tendencies and biases such as proximal cognition (short-term thinking; Björkman, 1984), comparative optimism (the belief that others are more at risk for an event than the self; Shepperd et al., 2002), and motivated reasoning (Sinatra et al., 2014). With such biases at play, individuals may fall prey to the view that global climate change is not personally relevant, thereby thwarting the development of strong attitudes.

Even individuals who believe strongly in climate change may not necessarily view it as a serious problem if they think it will bring about both good and bad outcomes (Krosnick et al., 2006). For example, individuals may believe that while increases in temperature may lead to flooding in distant places in the world, it also may produce more opportunities to enjoy days out at the beach, thus leading to mixed or ambivalent attitudes (Cacioppo et al., 1997) about global climate change insofar as their attitudinal structures may involve both positive and negative components. Ambivalent attitudes are a type of weak attitude, and operate like any other weak or undeveloped attitude – they are generally not highly vested, less predictive of subsequent behavioral intentions and behavior, and more susceptible to the effects of persuasive communication (Armitage and Conner, 2000; Crano et al., 2019). As such, weakly formed or ambivalent attitudes toward climate change are unlikely to translate into strong support for policies and to incite behavior to mitigate the effects of global climate.

In sum, due to the complexity of climate science and the political controversy surrounding human-induced climate change, we surmise that the issue of climate change is more aligned with an attitudinal formation than attitude change context for the public. This may be particularly the case for Republicans who tend to be much less concerned about climate change than Democrats (Pew Research Center, 2020). In the current research, the attitude formation versus change context was operationalized by the strength of attitudes, which was defined through attitudinal importance (see Krosnick and Smith, 1994). Attitudinal importance refers to the extent to which an attitudinal object is personally important to an individual – the less important, the weaker the attitude. Next, we discuss CCM’s predictions concerning attitude formation below.

### Minority and majority sources

In attitude formation, the CCM predicts the counterintuitive postulate that a minority message source has a persuasive advantage over a majority source, and focal attitude change is immediate (Crano and Seyranian, 2009). Why might this be the case? Crano and colleagues suggest that minorities can capture the attention of the audience because they are relatively rare (Crano, 2000) and their position is not normative. Thus, minorities may spark curiosity as people wonder why a small group would go out on a limb to advance their position, risking ridicule and ostracism. With increased curiosity elicited by the minority, its message is more likely to be processed and less likely to be counterargued because of weak pre-existing attitudes. The result is an immediate shift in attitudes or judgments in line with the minority position. Research has generally supported this prediction in attitude formation contexts (Crano and Hannula-Bral, 1994; Martin and Hewstone, 2003; Baron and Bellman, 2007). However, little research has tested the persuasive impact of minority and majority sources on global climate change attitudes. In clear attitude formation contexts (like for Republicans), and in line with the CCM, we expect stronger persuasive effects resulting from exposure to a minority than a majority source. But the persuasion landscape is more complex than this prediction. To fully account for influence processes in attitude formation, we must consider how influence effects are altered as a function of the nature of the task and the ingroup or outgroup status of the message source.

### Ingroup and outgroup source effects on subjective versus objective judgments

Besides majority and minority status, it also is important to consider whether the source of the message shares common group membership with the message recipient. Research has yielded mixed findings regarding the influence potential of ingroup and outgroup majority and minority sources. Some studies have found that outgroup minorities can influence the majority (e.g., Pérez and Mugny, 1987; Volpato et al., 1990) and others have shown that outgroup minorities influence less than ingroup minorities (e.g., Nemeth and Wachtler, 1973; Clark and Maass, 1988; David and Turner, 1996). The CCM can help regularize this literature by predicting variations in the persuasive power of ingroup or outgroup, majority or minority sources in attitude formation contexts as a function of the subjective or objective nature or the judgment in question. Subjective judgments involve issues where there are no verifiable right or wrong answers and judgments reflect matters of preference or palate. In contrast, objective judgments have verifiable consensually agreed-upon answers. Gorenflo and Crano (1989) showed that individuals tended to confer with similar others (ingroups) on subjective judgments because they are assumed to be more like the self and to share their worldviews, beliefs, and values. Individuals preferred to confer with dissimilar sources (outgroups) on objective judgments: conferring with ingroup sources might lead to the same wrong answers, as similar individuals are likely to share the same biases (Laughlin and Ellis, 1986; Laughlin, 1988). Outgroup sources are less liable to hold the same biases and may have valuable information to share. Thus, surveying diverse perspectives may increase the likelihood of attaining correct solutions to objectively verifiable questions.

Crano and Hannula-Bral (1994) extended Gorenflo and Crano (1989) study by testing the influence of majority and minority, ingroup and outgroup sources. Based on their responses on a minimal groups task, students were told their group was composed of either a majority or minority of students. Ingroup and outgroup sources were not operationalized in terms of the membership of participants in a particular group (e.g., students versus professors), but rather was based on whether students shared the same majority or minority status as a result of the minimal groups task. In Crano and Hannula-Bral (1994) experiment, participants were asked to answer difficult questions on computer screens. Participants were extremely unlikely to have had previous experience with these questions, thereby assuring an attitude formation context. They were told these questions were either objective (i.e., had a correct answer) or subjective (i.e., intuitions had to employed to formulate an answer). Then, students were ostensibly set up with a partner who was either a member of the majority or minority, therefore sharing either ingroup or outgroup status with the participant. In fact, the “partner” was a computer program designed to provide a consistent directional influence on the participant. Results supported CCM predictions. On objective tasks, majority group participants were more influenced by a minority partner (an outgroup). Minority group participants were more influenced by majority sources (an outgroup), but as expected, the effect was not as pronounced because majorities were not theorized to command as much attention as a minority source. For subjective tasks, minority group members relied more on the minority source (an ingroup). Although majority group members should be more likely to rely on majority partners (ingroup), results showed that they were equally likely to rely on either majority or minority partner. The authors argued this was because the minority was able to capture more attention than the majority, thereby evening the persuasive playing field.

Although this study relays important insights into the influence of majority and minority sources with objective and subjective tasks, it did not manipulate ingroup and outgroup social identities and test predictions concerning the influence potential of majority and minority factions. As Crano (2010, pp. 62–63) indicated, “whether or not the minority need be ingroup was not definitively answered in Crano and Hannula-Bral’s research. The minimal groups procedure was engaged to create ingroup and outgroup allegiances, but this treatment operated in opposition to participants’ long-standing and strong identification as students of the same university, a university that students have traditionally employed as a strong source of social identity.” The current study directly addresses this issue. It also provides a different test of CCM predictions by testing an alternate operationalization of subjective versus objective tasks, which has important implications for climate change discourse.

## Attitudes and plausibility

In the current research, we operationalized subjective tasks as attitudes on global climate change. Attitudes are evaluative tendencies (favor or disfavor) that are more difficult to verify or falsify with objective information (Eagly and Chaiken, 1993). As such, they are more subjective and may be influenced by social identifications such as political party allegiance. Attitudes about global climate change provide an interesting arena to tap into subjective evaluative responses to objective, scientific data and policy implications.

*Plausibility* concerns one’s conceptual knowledge. It represents a tentative epistemic judgment about the potential validity of incoming information. Unlike attitudes, plausibility is not valenced. It is a judgment about whether an assertion is right or wrong. Plausibility judgments are fundamentally different from probability judgments because individuals may consider two alternative explanations about a specific phenomenon as plausible (e.g., dinosaurs became extinct due to a climate change or an asteroid hitting earth or both) (Lombardi et al., 2016). Such judgments violate basic probabilistic rules. Plausibility judgments are less precise than probability judgments, but still maintain a fundamentally objective orientation. Plausibility judgments are often tentative. However, when made through more explicit and reasoned evaluations (e.g., evaluations of explanations based on scientific evidence), plausibility judgments are more objective (Sinatra and Lombardi, 2020).

To measure objective perceptions in the present research, participants were asked to provide plausibility judgments regarding climate change. Lombardi et al. (2016) assert that plausibility judgments are activated automatically when individuals are faced with competing explanations (e.g., an incoming message about climate change and background knowledge about weather and climate). Such judgments are most relevant when a gap exists between what laypersons and scientists find plausible. For example, many individuals consider assertions that climate change is caused by human actions (Lombardi and Sinatra, 2012; Lombardi et al., 2013), but the scientific literature on climate change shows greater than 99% consensus that human activities are the primary cause of current climate change (Cook et al., 2016; Lynas et al., 2021).

Recent evidence shows that individuals’ perceptions of sources are related to plausibility perceptions (Lombardi et al., 2014): When individuals judged a message source trustworthy, they deemed human-induced climate change appeals as more plausible. Trustworthiness and other characteristics may contribute to cognitions about the validity of a source (Schroeder et al., 2008), which through dynamic interplay, may act as an automatic and unconscious step in the formation of plausibility judgments (Lombardi et al., 2013). This also may be true about ingroup and outgroup minority and majority sources, yet to our knowledge, little research has examined this possibility (but see Lalot et al., 2019). For example, what happens when individuals are exposed to an outgroup source who endorses the position of an ingroup minority? The CCM provides insights into such questions, which are examined in the pages that follow.

## Overview of the research and hypotheses

The goal of the current research was to test predictions derived from the CCM for the issue of global climate change. This research also sought to contribute to our understanding of the CCM and plausibility by testing whether membership in and identification with a particular social identity (e.g., Democrat or Republican partisans) influenced persuasion attempts of majority and minority factions within these groups.

Although it was surmised that global climate change represented an attitude formation context for all students in the sample, it was specifically predicted that global climate change Republicans would report weaker (less well developed) pre-test attitudes about global climate change than Democrats (H1a). It was also hypothesized that Democratic social identification (but not Republican) would be positively related to both pre-test climate change attitudes and attitudinal strength (H1b).

Building on the findings of the pretest, we continued on to test the postulates of the CCM regarding the hypothesized dynamics of attitude formation for the Republican sample. The Democrat sample was hypothesized to hold relatively stronger attitudes about global climate change, therefore allowing us to investigate attitudinal *polarization* of attitudes after exposure to a pro-attitudinal message.

The current study examined shifts in attitudes and plausibility based on the following sources – ingroup majority, ingroup minority, outgroup majority, and outgroup minority–after exposure to a text arguing in favor of the acceptance of global climate change. To operationalize ingroup and outgroup minority and majority sources, we employed real world social identities – Democrat and Republican American political party identities. Republican and Democratic participants were randomly assigned to conditions that attributed the global climate change text to a Republican or Democratic author. Because it is established that Democrats are more likely than Republicans to express beliefs in line with the scientific consensus on global climate change (McCright and Dunlap, 2011), the text featured relatively prototypical content for a Democrat source (majority position) and non-prototypical content (minority position) for a Republican source.

In conditions where the text was attributed to a Democrat source, the message would be viewed as endorsing a majority position within the group. If the individual exposed to the text also was a Democrat, the source would be considered an *ingroup majority source*. In contrast, if the text was read by a Republican, the source would be considered an *outgroup majority source*. In conditions when the same pro-global climate change text was attributed to a Republican, the text would be construed as a minority position because Republicans tend to express skepticism regarding climate change. If a Republican read the text, the source would be perceived as an *ingroup minority*, whereas if a Democrat read the text target, the source would be seen as an *outgroup minority* (see Table 1). Participants provided ratings of their attitudes and plausibility judgments before and after reading the message. In this way, changes in participants’ responses could be measured as a function of source type and text exposure.

**TABLE 1:** Source, common group membership, and majority and minority status.

	Participant perception of source	
Source	Ingroup	Outgroup
Majority	Democrat participant views source as ingroup majority	Republican participant views source as outgroup minority
Minority	Republican participant views source as ingroup majority	Democrat participant views source as outgroup minority

In line with the CCM, we predicted that the subjective (attitudinal) or objective (plausibility) nature of the task would reveal disparate results. For attitudes concerning global climate change (subjective task), we predicted that individuals exposed to an ingroup source would show more favorable attitudes toward global climate change (H2). More specifically, Republicans with weak attitudes would become more favorable to global climate change when exposed to an ingroup minority position (a Republican source) advocating for the acceptance of the global climate change communication (H3). However, Democrat participants (hypothesized to have stronger attitudes) are predicted to shift their attitudes to align with the ingroup majority (i.e., Democratic) source, thereby evidencing more extreme attitudes (polarization) (H4).

The persuasion landscape changes for objective judgments. We predicted plausibility (or objective) judgments concerning global climate change would increase when individuals were exposed to an outgroup (H5), but not an ingroup source, because an outgroup source is dissimilar to the self. A dissimilar (outgroup) position is more appealing on objective judgments because diverse perspectives may increase the likelihood of attaining the correct solution (Laughlin and Ellis, 1986). It also was predicted that minority or majority outgroup sources would positively increase plausibility judgments (H6 and H7). However, the outgroup majority is predicted to induce larger changes (in terms of effect size) in plausibility than outgroup minorities (H8). The reasoning for this prediction lies in the idea that the participant would be persuaded by the source or position most dissimilar to the self. In this case, for global climate change, the most dissimilar position to the self would not be the outgroup minority (i.e., a Democrat exposed to a Republican source who accepts global climate change) because the position of the text is likely in line with the target’s position. Instead, the most dissimilar position to the self in this case would be the outgroup majority (i.e., a Republican exposed to a Democrat who accepts global climate change) because the position is more likely to contradict the target’s position. The outgroup minority (Republican) source in this case would still be persuasive as its position aligns with the pre-existing attitude of the Democrat participant, who largely accept global climate change.

## Method

### Participants

Two hundred ninety-eight students from a university in California volunteered to participate in the study via the Psychology Department’s subject pool. Participants were required to be 18 years of age to participate and received extra course credit for their participation. Seventy-three percent of participants were Democrats (*n* = 119) and 27% (*n* = 43) were Republican. All participants indicating that they were Independents (*n* = 118) or “other” political affiliation (*n* = 20) were excluded from the analyses as we were interested only in individuals with clear and consensual social identities and political platforms in the United States – that is, Republicans and Democrats. Independents, who comprised of a large number of the sample, were excluded from analysis (*n* = 118) because they: (a) vary politically on the liberal-conservative continuum, and thus (b) their stance on global climate change is not consistent. Students indicating “Other” political group (e.g., Peace and Freedom Party) also were excluded from analyses for similar reasons, and due to small sample size (*n* = 20). As a result, the final sample was comprised of 162 Democrat and Republican participants. Participants were between 18 and 38 years of age (*M* = 20.3, *SD* = 2.06), with 9% freshmen, 26% sophomore, 38% juniors, and 27% seniors. The sample was predominantly White (49%) and Asian American (31%), with 4% African Americans, 9% Hispanics/Latinos, 5% mixed ethnic groups, and 2% other. Due to human error, data for participants’ gender was not collected. However, it can be surmised that a ratio of approximately 1 (man) to 3 (women) existed in the current study based on gender data obtained from another study conducted by the first author with the same subject pool population during the same time period.

## Materials

### Climate change text

Participants were asked to read a text on climate change that argued against the idea that climate change was uncertain and advocated specific policies to ameliorate future climatological impacts. The first two-thirds of the text (592 words in length) discussed the scientific evidence for current climate change and the evidence connecting human activities to current climate change. The last third of the text (314 words in length) discussed a policy proposal to mitigate future climate change. The text was adapted from an editorial that appeared in the *New York Times* on May 10, 2012. The editorial was written by Dr. James Hansen, a noted climatologist and global climate change activist, who recently headed NASA’s Goddard Institute for Space Studies. We made slight modifications to the original editorial to increase the ambiguity about the text’s author. The text was just below the readable level for university students; the Flesch-Kincaid grade level was at a 9.8 (9th–10th grade) and the Flesch reading ease index was 53.6 (10th–12th grade). For the complete communication, see the Supplementary Appendix.

### Attitudes on global climate change

Attitudes concerning global climate change were measured both before and after participants read the editorial. The measure asked participants to rate their attitudes on global climate change on four semantic differential scales with the following end poles: urgent-not urgent, harmless-harmful, not frightening-frightening, not serious problem-serious problem. All items were rated on 1–7 scales. Cronbach’s alpha was acceptable for both pretest (α = 0.86) and post-test (α = 0.91) attitude measures.

### Attitude strength about global climate change

A three-item *attitude strength* scale adapted for global climate change from Boninger et al. (1995) asked participants to report the extent to which global climate change: (a) “does not mean anything-means a lot” to them; (b) is “unimportant-important,” and (c) whether they “do not care at all-cared a lot” about global climate change (*α* = 0.87). The items were rated on seven-point semantic differential scales. Participants who scored lower on this measure indicate weaker attitudes concerning global climate change.

### Plausibility perceptions of climate change

To measure participants’ plausibility perceptions of climate change, we administered the Plausibility Perceptions Measure (Lombardi and Sinatra, 2012), before and after reading the editorial. This instrument has eight statements about climate change based on the latest summative report produced by the United Nations’ Intergovernmental Panel on Climate Change (2007). The measure’s statements matched the major conclusions made in the report. For example, “Human influences on climate extend beyond average global temperature to other aspects, such as rising sea levels and widespread melting of snow and ice” (Intergovernmental Panel on Climate Change, 2007, p. 2). Participants rated each statement on a rating scale from 1 to 10 scale (1 = greatly implausible or even impossible to 10 = highly plausible). Cronbach’s alpha for the Plausibility Perceptions scale was excellent for both pretest (α = 0.93) and post-test (α = 0.95).

### Political party and social identification

All participants were asked to complete their political party affiliation prior to reading the global climate change text (Democrat, Republican, Independent, or Other). Then, Republican (α = 0.94) and Democrat participants (α = 0.95) indicated their identification with their political party by completing an 11-item social identification scale developed by Hains et al. (1997).

### Procedure

Students volunteered to participate in a study on “Global Climate Change” in exchange for course credit through the psychology department’s subject pool website. They completed the experiment online on their own computers via computerized survey software (Qualtrics). As they opened the link to the survey, participants completed various pretest items including their attitudes on global climate change, their attitude strength about global climate change, and the plausibility perceptions of climate change scale. Then, participants indicated their political affiliation (Democrat, Republican, Independent, or Other) and rated the extent to which they identified with their political party. Participants completed these measures prior to reading the editorial that manipulated ingroup or outgroup status of the source. The measures were designed to render participants’ own political party affiliation salient and ensure they would view the Republican or Democrat source as either ingroup or outgroup. Next, participants read the editorial on global climate change. Prior to reading the text, participants were randomly assigned (via Qualtrics) to receive varied instructions regarding the political affiliation of the message source. Participants were led to believe that the source and author of the message was either a Republican or Democrat. After reading the editorial, participants completed a variety of dependent measures including post-attitude and plausibility measures of global climate change. Once these items were completed, all participants were debriefed through a written online debriefing statement. All in all, it took students 25–45 min to complete the experiment.

## Results

### Descriptive statistics and correlations

Table 2 shows the overall means, standard deviations, and bivariate Pearson’s correlations for the variables (attitudes and plausibility perceptions), on both pre- and post- measurements. Correlations between pre and post-attitudes [*r* (158) = 0.75, *p* < 0.001], and between pre and post-plausibility ratings [*r* (151) = 0.85, *p* < 0.001] were statistically significant. Associations between attitudes and plausibility also were significant (all *r*s > 0.49–0.71, *p* < 0.001), indicating that attitudes and plausibility are disparate, but related constructs, as hypothesized.^1^

**TABLE 2:** Bivariate correlations and descriptive statistics for the study variables.

Measure	1	2	3	4
1. Attitudes pre	–			
2. Attitudes post	0.75**	–		
3. Plausibility pre	0.62**	0.70**	–	
4. Plausibility post	0.49**	0.71**	0.85**	–
*M*	5.55	5.87	8.01	8.29
*SD*	1.09	0.97	1.46	1.46

The possible score range was 1 to 7 for the attitudes measure, and 1 to 10 for plausibility measure, **p < 0.01.

### Attitude formation context

To test whether Republicans had weaker attitudes about global climate change than Democrats (H1a), an independent samples *t*-test was conducted with political party as the independent variable (Democrat or Republican) and pretest attitude strength as the dependent variable. In line with expectations, Republicans (*M* = 4.33, *SD* = 1.08) had weaker attitudes than Democrats (*M* = 5.54, *SD* = 0.97), *t*(160) = −6.80, *p* < 0.001.^2^ In line with hypothesis 1b, Pearson correlations also revealed that Democratic social identification was associated with pre-test attitudes [*r*(117) = 0.22, *p* = 0.01] and (marginally) related to pre-test attitudinal strength [*r*(117) = 0.16, *p* = 0.07]. However, Republican social identification was not correlated with pretest attitudes on climate change [*r*(43) = −0.03, *p* = 0.85] or pre-test attitude strength [*r*(43) = 0.05, *p* = 0.73]. This suggests that global climate change aligns more with an attitude formation context for Republican participants than Democrats.

### Attitudes

We conducted a 2 (ingroup, outgroup) × 2 (majority, minority) × (pre-attitudes, post-attitudes) repeated measures mixed factorial analysis of variance (ANOVA) to examine source effects on global climate change attitudes. The between-subjects factors were ingroup/outgroup status and majority/minority group position. The within-subjects factor was time (pre and post-attitudes). This repeated measures ANOVA did not meet the homogeneity of the variance-covariance matrices assumption (Box’s *M* = 32.8, *p* < 0.001). Therefore, the more stringent Pillai’s criterion was used in significance testing (Tabachnick and Fidell, 2007). A three-way interaction between pre-post attitudes, group status (ingroup or outgroup), and majority or minority position within group was statistically significant, *F*(1,156) = 3.95, *p* = 0.05, η*_*p*_^2^* = 0.03.

Follow-up tests revealed no significant pre to post-attitude differences for outgroup sources, *F*(1,73) = 0.34, *p* = 0.56. In support of hypothesis 2, the tests did reveal significant differences in pre and post-attitudes for ingroup sources, *F*(1,83) = 6.51, *p* = 0.01, η*_*p*_^2^* = 0.07 (see Table 3). In line with hypotheses 3 and 4, people exposed to both ingroup *minority* and ingroup *majority* sources became more favorable toward climate change. The shift for Republicans exposed to an ingroup minority source (a fellow Republican who accepts climate change) had a large effect size, *F*(1,23) = 16.0, *p* = 0.001, η*_*p*_^2^* = 0.41. The atitudinal shift for Democrats exposed to the ingroup majority (fellow Democrats) had only a moderate effect size, *F*(1,60) = 14.30, *p* < 0.001, η*_*p*_^2^* = 0.19.

**TABLE 3:** Score means for attitudes about climate change.

	Ingroup source		Outgroup source	
	Minority status (Republican views source as Republican)	Majority status (Democrat views source as Democrat)	Minority status (Democrat views source as Republican)	Majority status (Republican views source as Democrat)
Pre-attitudes about climate change (*N*)	4.58* (1.16) (24)	5.94** (0.95) (61)	**5.76** (0.90) (57)	**4.83** (0.96) (18)
Post-attitudes about climate change (*N*)	5.25* (1.01) (24)	6.21** (0.68) (61)	**5.98** (0.91). (57)	**5.18** (1.21) (18)

The possible score ranges for pre- and post-attitudes were 1 to 7. Mean scores are in bold and standard deviations are in parentheses. Attitude shift from pre to post for outgroup source was not significant, therefore, additional analyses for outgroup minority and majority status pre to post was not warranted. * Attitude shift from pre to post was significant (p = 0.001), with a moderate effect size (η_p_^2^ = 0.19). ** Attitude shift from pre to post was significant (p < 0.001), with a large effect size (η_p_^2^ = 0.41).

### Plausibility perceptions

We also conducted a repeated measures 2 (ingroup, outgroup) × 2 (majority, minority) × (pre-plausibility, post-plausibility) mixed factorial analysis of variance (ANOVA) to examine source effects on plausibility perceptions of climate change. Like our analysis of attitudes, the between-subjects factors were ingroup/outgroup status and majority/minority position, and the within-subjects factor was time (pre and post-plausibility ratings). This repeated measures ANOVA did not meet the homogeneity of the variance-covariance matrices assumption, with Box’s *M* = 21.25, *p* = 0.02. Therefore, the more stringent Pillai’s criterion was used in significance testing (Tabachnick and Fidell, 2007). A three-way interaction between pre-post plausibility, group status (ingroup or outgroup), and majority or minority position within group was statistically significant, *F*(1,149) = 5.02, *p* = 0.03, η*_*p*_^2^* = 0.03. Follow-up tests did not reveal any significant pre to post-plausibility differences for ingroup sources, *F*(1,82) = 0.77, *p* = 0.38, η*_*p*_^2^* = 0.009. In line with hypothesis 5, follow-up tests did reveal significant differences in pre and post-plausibility for outgroup sources, *F*(1,67) = 5.64, *p* = 0.02, η*_*p*_^2^* = 0.08 (see Table 4). In support of hypotheses 6 and 7, individuals exposed to either an outgroup minority or outgroup majority source shifted in their plausibility perceptions. The shift for Republicans exposed to an outgroup majority (Democrat source) reflected a large effect size, *F*(1,16) = 8.72, *p* = 0.009, η*_*p*_^2^* = 0.35. The shift for Democrats exposed to outgroup minority (Republican source) had only a moderate effect size, *F*(1,51) = 7.31, *p* = 0.009, η*_*p*_^2^* = 0.13. This supports hypothesis 8, in which the outgroup majority was predicted to induce larger effect size changes in plausibility than outgroup minorities.

**TABLE 4:** Score means for plausibility perceptions of climate change.

	Ingroup source		Outgroup source	
	Minority status (Republican views source as Republican)	Majority status (Democrat views source as Democrat)	Minority status (Democrat views source as Republican)	Majority status (Republican views source as Democrat)
Pre-plausibility on climate change (*N*)	**7.10** (1.30) (24)	**8.45** (1.20) (60)	8.21* (1.26) (52)	6.71** (1.92) (17)
Post-plausibility on climate change (*N*)	**7.54** (1.57) (24)	**8.70** (1.26) (57)	8.45* (1.26) (52)	7.43** (1.86)** (17)

The possible score ranges for pre- and post-plausibility were 1 to 10. Mean scores are in bold and standard deviations are in parentheses. Plausibility shift from pre to post for ingroup source was not significant, therefore, additional analyses for ingroup minority and majority status pre to post was not warranted. * Plausibility shift from pre to post for outgroup Republican sources was significant (p = 0.009), with a moderate effect size (η_p_^2^ = 0.13). ** Plausibility shift from pre to post was significant (p = 0.008), with a large effect size (η_p_^2^ = 0.35).

### Manipulation check

A manipulation check analysis was conducted to ensure participants in the Democrat or Republican source conditions correctly identified the source of the text as Democrat or Republican. A Chi-Square analysis showed 55% of participants in the Republican condition correctly identified the source of the text as a Republican. The remaining participants incorrectly identified the source as a Democrat (21%) or as having no political affiliation (24%). In the Democrat condition, 63% of participants correctly identified the source as a Democrat, whereas the remaining incorrectly identified the source as not possessing a political affiliation (30%), Republican (4%) or Independent (3%). Roughly half the participants in the various conditions correctly identified the text source. Given that the manipulation check item was placed at the very end of the survey, it is arguable that the source information was correctly absorbed by participants and influenced their attitudes and plausibility as shown in the earlier analyses, but by the time they reached the survey’s end, they failed to identify the source correctly. The significant and precisely expected pattern of results indicate the treatments operated as predicted, suggesting the invalidity of the single-item manipulation check (Crano et al., 2015).

## Discussion

The current study examined whether specific predictions derived from the CCM increased the persuasive effects of a science-based global climate change policy appeal. Our goal also was to examine whether the CCM’s predictions for attitude formation also held when real-world social identities formed the basis of ingroup and outgroup status (e.g., Democrat or Republican) rather than groups derived from minimal groups tasks (Crano and Hannula-Bral, 1994) or other group-formation methods of low mundane realism. The results showed that Republicans held weaker attitudes about global climate change than Democrats. As hypothesized, Republican participants’ responses strongly supported the predictions of the CCM for attitude formation contexts. The findings suggested that source effects differentially influence subjective and objective tasks. For more subjective matters such as attitudes, our results for both Republican and Democrat participants indicated that individuals were more influenced by common ingroup membership. Although both majority and minority ingroup sources significantly produced shifts in attitude, the relative salience of the ingroup minority source in the attitudinal formation context contributed to larger shifts in attitudes than the ingroup majority source. Given that the Democrats held stronger attitudes about global climate change, the ingroup majority source moderately polarized their attitudes from pre to post.

For more objective matters such as the plausibility of climate change, outgroup sources held a clear persuasive advantage. Republican participants were significantly persuaded by dissimilar sources who were members of the outgroup (Democrats) as were Democrats’ plausibility judgments, which became more polarized when exposed to an outgroup minority (Republicans). In other words, participants preferred sources whose orientation was different from their own so that they were more likely to triangulate on the proper solution (Crano and Seyranian, 2009). Although both outgroup minorities and majorities increased plausibility perceptions of global climate change, larger shifts were produced for Republicans who were exposed to an outgroup majority than Democrats who were exposed to an outgroup minority. It should be noted that this finding was not consistent with Crano and Hannula-Bral (1994) research, where larger effects were obtained for the outgroup minority rather than the outgroup majority. Our results supported the idea that the most dissimilar source to the target exerted the most influence. In our case, although the outgroup minority might have had a salience advantage, the most dissimilar source to the self was *not* the outgroup minority (i.e., a Democrat exposed to a Republican source who accepts global climate change) but the outgroup majority (i.e., a Republican exposed to a Democrat who accepts global climate change). This was because the outgroup minority’s message (Republican arguing in favor of global climate change) was consistent with the position held by the ingroup majority (Democrats). However, the outgroup majority’s position (i.e., Democrat arguing in favor of global climate change) differed most markedly from the position held by the ingroup (Republicans). In this way, individuals may have been most persuaded by the most dissimilar source. An alternate explanation is also feasible. With objective tasks, while the minority retains the ability to capture attention, individuals may be more persuaded by the outgroup majority position because they are seeking consensus to settle on a correct solution (Mackie, 1987). As Mackie (1987) suggests, “the majority is seen as reflecting objective reality” (p. 42). Future research should attempt to replicate the findings for objective tasks with an alternate topic and test these competing explanations – seeking maximum dissimilarity or social consensus – to clarify outgroup majority effects.

The results also contribute to the literature on plausibility judgments. Specifically, findings support the role of source validation and evaluation on plausibility judgments (Maier and Richter, 2012; Lombardi et al., 2014), with ingroup/outgroup and majority/minority status resulting in differences among plausibility perceptions. With plausibility potentially playing an important role in reconstructing knowledge to be consistent with scientifically accepted understandings (Lombardi et al., 2018; Heddy et al., 2022), further research in this area is warranted.

### Implications for global climate change discourse

One of the goals of the current research was to test insights from the social influence literature in hopes of ascertaining effective message strategies for global climate change discourse. Despite some study limitations noted below, the research suggests the CCM has important insights that can augment the persuasive appeal of important scientific issues like global climate change messages (Crano and Alvaro, 1998; Crano, 2000, 2012; Crano and Seyranian, 2009). First, it is important to determine the attitude formation versus attitude change context of the audience for the topic at hand. In our Republican sample, global climate change was viewed as an attitude formation context, which altered the persuasion landscape for different sources. For the Democrat sample, global climate change attitudes were strongly held, thereby creating the potential for attitudinal polarization. Second, the communicator should seek to render salient a valued social identity for the message target (e.g., political party identity) and implicate this social identity in the influence attempt with appropriate source information (ingroup or outgroup). Determining whether to stress an ingroup or outgroup source will depend on the task at hand, which brings us to the third insight. It is important to decide the goal of the science communication. Is the goal to shift attitudes about the issue at hand (subjective task) or to increase plausibility judgments pertaining to scientific evidence (objective task)? Depending on the science communicator’s goal – whether to influence targets regarding a subjective or objective task – different source types are most optimal. If the goal is to influence attitudes, framing the message as either from an ingroup minority or majority source may prove effective. However, larger influence effects may be obtained by stressing that the source of the message is an ingroup minority. If the goal is to increase perceptions of the plausibility of climate change (and potentially induce conceptual change and deeper conceptual understanding of a topic), framing the message from an outgroup minority and majority source may prove influential. Yet, larger influence effects will likely be obtained by framing the message from an outgroup majority source. Overall, our research suggests that there is no “one-size fits all” approach that can influence everyone. A variety of variables that include source, target, and task variables need to be considered to optimize the persuasive impact of climate change discourse.

### Limitations

Various limitations of the study should be noted. First, we sampled from a student population in the Western United States. As such, additional research is needed on more representative national and international samples to allow more definitive conclusions about messaging strategies for global climate change. This also will help establish whether global climate change is more an attitude formation or attitude change context for Republicans and Democrats. We surmise that the attitude formation versus attitude change context of global climate change is important to consider and may differ based on regions and countries.

Second, some of our participants did not appear to note source information (see manipulation check analyses) – which is a common finding in social psychological research. Future research should be attentive to the location of source manipulation items in survey as it has the potential to influence error rates.

Third, our sample excluded independents and those with “other” political affiliations (e.g., Peace and Freedom Party) due to the methodological necessity of including only individuals with clear and consensual social identities. However, Independents and other political affiliations constituted almost half the current sample, and 4 in 10 Americans identify as Independent (Pew Research Center, 2019). Therefore, future research should extend the current study to examine how independents and other political affiliations fare with different source types in the persuasion landscape. One suggestion along these lines may be to move away from employing categorical self-reports of political party preferences when the interest is to include Independents in the sample. Future research might use a continuous measure of the liberal-conservative continuum, which may also capture the views of independents, but such a study, or studies, has yet to be designed.

Fifth, this study does not explicitly manipulate the majority or minority status as is the case in much minority influence research, but instead, ingroup and outgroup minority or majority status is inferred by the prototypical position on the issue at hand in the group in question: i.e., where prototypical Republicans or Democrats stand on climate change, the source and reader’s social identity identification, and the extent that the source represents a prototypical (majority) or less prototypical (minority) position in their own group. Therefore, if a Democrat was reading a message from a Republican source (outgroup) advocating climate change policy initiative (minority position in the Republican party), it would be considered an outgroup minority source. This alternate operationalization of minority and majority status is arguably a more ecologically valid way of how majority and minority status plays out with social identity dynamics in persuasion contexts outside the laboratory. However, it is acknowledged that in the current experimental design, majority and minority status also could be seen as confounded with political affiliation and future research should strive to disentangle these effects, while maintaining the ecological validity of the research. A simple solution might involve a parallel study with an issue (or issues) on which the positions were opposite those used in the current research. This would maintain experimental and mundane realism, but would not unlink the source-message relationship. A replication of results obtained in the present study, employing issues of opposite valence to contending groups, would help bolster confidence in the stated implications of the findings, even though not undoing the linkage of source and prototypical issue position. Such a study would preserve ecological validity at the expense of methodological purity, and hopefully motivate others to seek more standard and ecologically valid approaches.

A final limitation is that our study did not seek to discern the difference between weak, ambivalent, or non-existent attitudes regarding global climate change. This distinction may have important practical implications for persuasion and warrants future research.

## Conclusion

Overall, this research underscores that important insights from the field of social influence can optimize the effectiveness of communications about scientific issues such as global climate change. Such understanding is essential for developing effective messages to mobilize actions toward policies aligned with scientific consensus (Lombardi, 2022). The CCM provides a promising avenue to guide such framing efforts for science communication – it highlights the complexity of the persuasive communication process and the importance of considering attitude formation and attitude change contexts, the message source, target, and task variables.

## Data availability statement

The raw data supporting the conclusions of this article will be made available by the authors, without undue reservation.

## Ethics statement

The studies involving human participants were reviewed and approved by the Institutional IRB. The patients/participants provided their written informed consent to participate in this study.

## Author contributions

VS, DL, and GS conceptualized the study and methodology. VS collected the data and wrote the manuscript draft. VS and WC analyzed and interpreted the data. DL, GS, and WC edited the manuscript draft. All authors contributed to the article and approved the submitted version.

## Footnotes

1 A factor analysis with varimax rotation (forced 2 factor solution) was conducted with all four of the pre-test attitudes items and all 8 of the pre-test plausibility items. Results showed that the two factors accounted for 70.65% of the variance. Factor 1 had all four attitude items comprising of 58.28% of the variance with factor loadings between 0.83 and 0.73. Factor 2 consisted of all eight of the plausibility items comprising of 12.31% of the variance with factor loadings between 0.83 and 0.67. This provides further evidence that although attitudes and plausibility are two related but different constructs.

2 We also ran exploratory analyses for pretest plausibility. Results showed that Republican participants (*M* = 6.95, *SD* = 1.55) rated climate change as less plausible than Democrats at pretest (*M* = 8.39, *SD* = 1.21), *t*(61.72) = −5.53, *p* < 0.001 (equality of variance is not assumed). Plausibility at pretest was also not significantly correlated with Republican social identification, *r*(39) = −0.10, *p* = 0.51, but Democratic party identification was significantly correlated to plausibility of climate change at pretest, *r*(117) = 0.29, *p* = 0.002.
