# Supplementary material for: Optimizing climate change communication: Context Comparison Model method
Source: Front Psychol. 2022 Aug 3;13:897460. doi: 10.3389/fpsyg.2022.897460 (PMC9382098; doi:10.3389/fpsyg.2022.897460)
Supplement: Supplementary file 1 [file Image_1.pdf]

Appendix

EDITORIAL

Global warming isn't a prediction. It is happening. That is why I was so troubled to read a recent interview with an American Senator in Rolling Stone. He said that Canada would exploit the oil in its vast tar sands reserves "regardless of what we do."

If Canada proceeds and we do nothing, it will be game over for the climate.

Canada has extremely large quantities of fossil fuels obtained from tar sands. Tar sands are a type of unconventional petroleum deposit. The tar sands consist of loose sand containing naturally occurring mixtures of sand, clay, and water, saturated with a dense and extremely viscous form of petroleum. Canada's tar sands contain twice the carbon compared to regular drilling. If we fully exploit this new oil source and continue to burn conventional fossil fuels supplies, carbon dioxide in the atmosphere will exceed levels not seen since 2.5 million years ago, when sea level was at least 50 feet higher than it is now. This would assure that the disintegration of the ice sheets would accelerate out of control. Sea levels would rise and destroy coastal cities. Global temperatures would become intolerable. Twenty to 50 percent of the planet's species would be driven to extinction. Civilization would be at risk.

That is the long-term outlook. But near-term, things will be bad enough. Over the next several decades, the Western United States and the Great Plains from North Dakota to Texas will develop semi-permanent drought. Rain, when it does come, would occur in extreme events with heavy flooding. Economic losses would be incalculable. More and more of the Midwest would be a dust bowl. California's Central Valley could no longer be irrigated. Food prices would rise to unprecedented levels.

If this sounds apocalyptic, it is. This is why we need to reduce emissions dramatically. The U.S. government has the power to deny tar sands oil additional access to Gulf Coast refining, which Canada desires in part for export markets, and also to encourage economic incentives to leave tar sands and other dirty fuels in the ground.

The global warming signal is now louder than the noise of random weather. Extremely hot summers have increased noticeably. We can say with high confidence that the recent heat waves in Texas and Russia, and the one in Europe in 2003, which killed tens of thousands, were not natural events — they were caused by human-induced climate change.

We have known since the 1800s that carbon dioxide traps heat in the atmosphere. The right amount keeps the climate conducive to human life. But add too much, as we are doing now, and temperatures will inevitably rise too high. This is not the result of natural variability, as some argue. The earth is currently in the part of its long-term orbit cycle where temperatures would normally be cooling. But they are rising — and it's because we are forcing them higher with fossil fuel emissions.

The concentration of carbon dioxide in the atmosphere has risen more than 40% over the last 150 years. The tar sands contain enough carbon — 240 billion tons — to add an additional 30%. Oil shale—another dirty fossil fuel source that is found mainly in the United States—contains at least an additional 300 billion tons of carbon. If we turn to these dirtiest of fuels rather than finding ways to phase out our addiction to fossil fuels, there is no hope of keeping carbon concentrations below a level that would, as earth's history shows, leave our children a climate system that is out of their control.

We need to start reducing emissions significantly, not create new ways to increase them. We should impose a **Gradually Rising Carbon Fee** collected from fossil fuel companies. We

could distribute 100 percent of the collections to all Americans every month. The government would not get a penny. This market-based approach would stimulate innovation, jobs and economic growth, while also avoiding bigger government or having it pick winners or losers. Except for the heaviest energy users, American citizens would get more back than they paid in increased prices. Not only that, reductions in oil use resulting from the carbon fee would be nearly six times greater than oil supplies from Canada's tar sands. This makes the extracting oil from the tar sands superfluous.

But instead of placing a rising fee on carbon emissions to make fossil fuels pay their true costs and leveling the energy playing field, the world's governments are forcing the public to subsidize fossil fuels with hundreds of billions of dollars per year. This encourages a frantic stampede to extract every fossil fuel through mountaintop removal, tar sands and oil shale extraction, and deep ocean and Arctic drilling.

Our leaders must speak candidly to the public—which yearns for open, honest discussion explaining that our continued technological leadership and economic well-being demand a reasoned change of our energy course. History has shown that the American public can rise to the challenge, but leadership is essential.

The science of the situation is clear—it's time for the politics to follow. This is a plan that can unify conservatives and liberals, environmentalists and business. Every major national science academy in the world has reported that global warming is real, caused mostly by humans, and requires urgent action. The cost of acting goes far higher the longer we wait — we can't wait any longer to avoid the worst and be judged immoral by coming generations.
